# Access to malaria treatment in young children of rural Burkina Faso

**DOI:** 10.1186/1475-2875-8-266

**Published:** 2009-11-24

**Authors:** Maike Tipke, Valérie R Louis, Maurice Yé, Manuela De Allegri, Claudia Beiersmann, Ali Sié, Olaf Mueller, Albrecht Jahn

**Affiliations:** 1 Institute for Public Health, Ruprecht-Karls-University Heidelberg, Im Neuenheimer Feld 324, 69120 Heidelberg, Germany; 2 Centre de Recherche en Santé de Nouna (CRSN), Nouna, BP 02, Burkina Faso

## Abstract

**Background:**

Effective and timely treatment is an essential aspect of malaria control, but remains a challenge in many parts of sub-Saharan Africa. The objective of this study was to describe young children's access to malaria treatment in Nouna Health District, Burkina Faso.

**Methods:**

In February/March 2006, a survey was conducted in a representative sample of 1,052 households.

**Results:**

Overall 149/1052 (14%) households reported the current possession of anti-malarial medicine, which was significantly associated with urban area, literacy of household head, having young children, and high socio-economic status. Out of a total of 802 children under five years, at least one malaria episode was reported for 239 (30%) within the last month. Overall 95% of children received treatment, either modern (72%), traditional (18%) or mixed (5%). Most of the medicines were provided as home treatment by the caregiver and half of children received some type of modern treatment within 24 hours of the occurrence of first symptoms. Despite a recent policy change to artemisinin-based combination therapy, modern anti-malarials consisted mainly of chloroquine (93%). Modern drugs were obtained more often from a health facility in localities with a health facility compared to those without (60% vs. 25.6%, p < 0.001). In contrast, beside informal providers, volunteer community health workers (CHW) were the main source of modern medicine in localities without a health centre (28% vs. 3%, p < 0.001).

**Conclusion:**

Access to modern health services providing quality controlled effective combination therapies against malaria needs to be strengthened in rural Africa, which should include a re-investigation of the role of CHW 30 years after Alma Ata.

## Background

After decades of severe neglect, malaria control and even malaria eradication are now back to the global health agenda [1-3]. The Roll Back Malaria (RBM) partnership, which was initiated in 1998 by three UN agencies and the World Bank, has been very successful in raising global awareness and in multiplying existing funds [4,5]. One challenge is now to further increase and sustain funding over the next decades as outlined in the Global Malaria Action Plan of the RBM partnership [5].

These promising developments are already accompanied by a number of malaria control success stories in endemic areas [6-16]. However, most of these examples are from islands, fringes of endemic areas, smaller countries with significant external support, or from more developed countries outside sub-Saharan Africa (SSA). Whether progress can be expected to be comparable in the majority of SSA countries under the existing scenario of prevailing poverty, weak infrastructure and management capacity, unreliable donor funding, underused health services, and high malaria transmission intensity, remains doubtful [17,18].

Uptake of the main malaria control interventions--insecticide-treated bed nets (ITN) and artemisinin-based combination therapy (ACT)--is still low in SSA, with only one quarter of preschool children and pregnant women having been protected with ITN and only 3% of fever episodes having been treated with ACT in 2006, although numbers continue to increase [19-21]. Moreover, translating national policy changes such as changes in first-line therapy for malaria into practice remains a long and complicated process [22-26]. As a consequence, chloroquine and other ineffective mono-therapies have until very recently remained the treatment of choice for the majority of malaria cases in many SSA countries [21,27]. Most of the roughly one million annual malaria deaths occur in young children of rural areas [28-31], where a high proportion of cases rely on home-treatment with often substandard drugs from shops and markets [30,32-34]. In such areas and beside various socio-cultural and socio-economic factors influencing the pattern of health care-seeking behaviour, access to functioning modern health services and effective treatment regimens remains the major challenge to effective malaria control [30,35-44].

Burkina Faso, where malaria is holoendemic and represents a leading cause of infant mortality, adopted ACT as first-line treatment against malaria in 2005. However, the actual implementation of this national policy change took some time and ACT was only made available in governmental health services by the end of 2007. Here, the pattern of health care-seeking behaviour and access to modern health services and malaria treatment was analysed in young children of a rural district in Burkina Faso in 2006, before the change to ACT effectively took place.

## Methods

### Study area

The study was carried out in the Nouna Health District corresponding to the administrative province of Kossi in north-western Burkina Faso, a rural, multi-ethnic, dry-savannah orchard area of approximately 300,000 inhabitants.

Malaria is holoendemic in the area with peak transmission occurring typically between July and November during and shortly after the rainy season [45]. The 304 villages in the district were served by 25 primary health care facilities, including 24 health centres known locally as "*Centre de Santé et Protection Sociale*" in villages and one hospital located in the semi-rural town of Nouna, the district capital (see map in additional file 1). At the time of the study, the personnel of the health facilities charged patients for both consultation and drugs.

### Household sampling

The study was nested into a large cluster-randomized ITN trial in the Nouna Health District [46]. During this trial, a three-step sampling scheme was used to randomly select households for the survey. Briefly, the localities (villages and Nouna town) were first divided into 25 clusters corresponding to the geographical coverage area of the 25 primary health care facilities. Second, for each cluster, the locality with the health facility was included and an additional village was randomly selected. Finally, within each village 20 households were selected at random. In Nouna town, which includes approximately 7.5% of the district population, the sampling was done in every of its seven sectors and included 72 households (10 in sector one to six and 12 in sector seven). In total, 1,052 households from 50 different localities (49 villages plus Nouna town) were randomly selected, with 500 (47.5%) of them being located in a locality without a health facility.

The median population of villages without a health centre was 654 (range: 211-2,035) and the median distance by road to the nearest modern health facility was 7 km (range: 2-19 km). The median population of villages with a health centre was 1,783 (range: 878-7,322) and that of Nouna town 21,034.

### Household questionnaire survey

The questionnaire consisting of 20 mostly closed-ended questions inquired about the presence and origin of medicines in the household and about the occurrence and possible treatment of a malaria episode in a child under five within the last month (see survey questionnaire in additional file 2). Questions were asked about symptoms; response of the caregiver; medicines used as first, second and third treatment; the person deciding to administer treatment; origin of medicines; time between onset of symptoms and start of treatment; duration of treatment and outcome of illness episode. Furthermore household heads were asked to report medicines used against malaria that were present in their household. Medicines were defined as drugs taken internally and included both modern medicines and traditional treatments. Because of self-reporting, the drugs mentioned included actual modern anti-malarial medicines as well as other non-anti-malarial drugs. Modern medicines were defined as drugs that are commercially-traded based on one or more active ingredients. The interviewers requested to directly see the medicines and wrote down their name or described them as precisely as possible if not identifiable by name (e.g. round white tablets). Home treatment was defined as medicines administered at home by the caregiver as opposed to treatment recommended by individuals consulted outside of the home such as medical professionals or community health workers. For questions regarding malaria episodes in children under five, the mother or direct caregiver of the child was interviewed. The recall period was one month. The terms used for malaria were the French word "*paludisme" *and local word "*soumaya" *describing a malaria episode [47].

The questions were initially formulated in French. After a review with Burkinabé colleagues of the Nouna Health Research Centre (CRSN) and a pilot test, the interviewers, who spoke French, the local lingua franca Dioula and some local languages, were trained to administer the questionnaire so that the interview could be done in an appropriate language for each household. Interviews were carried out in the middle of the dry season (February/March) 2006.

The socio-economic status of the household was estimated by inventorying selected assets and computing their combined value by multiplying their number by their average market price. These assets included both farm animals (poultry, sheep, goats, cows, donkeys, etc) and other goods (radio, television, telephone, stove, refrigerator, vehicles, and plough).

### Quality control, data management and analysis

Every questionnaire was checked for completeness and consistency by field supervisors. Data from the paper questionnaires were entered by trained data entry clerks into Microsoft Access 11.0 through a data entry mask with a number of logical checks. At least one of the investigators was always present to answer questions, re-check randomly questionnaires and data entry for error and ensure overall quality control. Data analysis was done using Microsoft Excel, SPSS 12.0 (SPSS Inc, Chicago IL, USA) and SAS 9.2 (SAS Institute Inc., Cary, NC, USA). Measure of association was determined by using the chi square test in a univariate analysis and statistical significance was declared for *p *< 0.05.

### Ethical aspects

Ethical clearance was obtained from the ethic committee of the University of Heidelberg and the local ethic committee of the CRSN. Explicit oral consent to participate was obtained from the household head before each interview by asking if he agreed that the household took part in the study and stating that the interview could be stopped at any time and without any negative consequences.

## Results

### Demographic and socio-economic characteristics

The main ethnic groups represented in the survey were Bwaba (42%; 439/1052), Marka (29%; 302/1052), Peuhl (10%; 102/1052), Mossi (9%; 91/1052) and Samo (5%; 49/1052). Main religious groups were Muslims (50%; 527/1052) and Christians (37%; 385/1052). The majority of households (87%; 912/1052) were headed by a farmer, who was in most instances a man (95%, 1002/1052). The literacy rate (defined as the self-reported ability to read and write) among the male and female heads of household was 34% (340/1002) and 2% (1/50) respectively.

### Availability of medicine in the household

Overall 14% (149/1052) of households reported the current possession of anti-malarial medicine: 40% of these (59/149) had modern anti-malarials, 48% (71/149) modern anti-pyretics, 13% (19/149) other modern drugs, and 36% (53/149) traditional medicine. Antipyretics consisted of paracetamol (93%, 66/71) and aspirin (7%, 5/71) while modern anti-malarials consisted of chloroquine (86%, 51/59), quinine (7%, 4/59), amodiaquine (5%, 3/59), and sulphadoxine-pyrimethamine (SP) (2%, 1/59). Thus, less than 1% (8/1052) of all households had a stock of somewhat effective anti-malarials (quinine, amodiaquine, SP). Other modern drugs included antibiotics, anti-helmintics and anti-tussive drugs.

### Origin of modern medicine in the household

The origin of modern drugs found in households was varied and included official sources, such as pharmacies in health facilities and private pharmacies as well as the illicit market, namely shops or street/market vendors. Most modern drugs were obtained from a health facility (65%, 96/147) while in 23% (34/147) of households, they were obtained from the illicit market or from unknown sources. Amodiaquine, SP and quinine were only available in households located in villages with a health centre and had all been obtained either from health facilities or private pharmacies. In contrast chloroquine and anti-pyretics were available in all villages but still originated predominantly from official sources (80%, 41/51 and 72%, 51/71, respectively). Modern drugs in households were found more frequently in localities with a health facility compared to localities without (17%, 91/552 vs. 11%, 56/500, p = 0.01). Furthermore, modern drugs were purchased more frequently from a health facility in localities with a health facility compared to localities without (75%, 68/91 vs. 50%, 28/56, p = 0.004). 80% (12/15) of modern drugs purchased through the illicit market came from households in villages without a health centre and only in these villages were modern drugs (7%, 4/56) obtained from volunteer community health workers (CHW).

### Factors influencing the availability of modern medicine in the household

Table 1 shows how different household factors influenced the likelihood of having modern medicine in the household. Households located within 5 km of a health facility were slightly more likely to have modern medicine available at home compared to those located farther than 5 km away (11% vs. 7%, p = 0.07). A statistically significant difference was observed for households located in urban as compared to rural areas (18% vs. 9%, p = 0.01)

**Table 1:** Factors influencing the likelihood of presence of modern medicine in the household.

Factors	Total	N	%	OR	95% CI
**Health facility in locality**					
No	500	40	8.0%	1	
Health centre (in village)	480	48	10.0%	1.28	0.82-1.98
District hospital (in Nouna)	72	13	18.1%	**2.53**	**1.28-5.01**
**Urban vs. Rural**					
Rural	980	88	9.0%	1	
Urban	72	13	18.0%	**2.23**	**1.18-4.23**
**Distance from health facility**					
0-5 km	672	73	10.9%	1	
>5 km	380	28	7.4%	0.65	0.41-1.03
**Age of child**					
No child under five yr	813	67	8.2%	1	
0-11 months	45	9	20.0%	**2.79**	**1.29-6.03**
12-60 months	194	25	12.9%	**1.65**	**1.01-2.69**
**Household head literate**					
No	711	52	7.3%	1	
Yes	341	49	14.4%	**2.13**	**1.41-3.22**
**Asset-based indicator**					
1^st ^quartile (poorest)	263	11	4.2%	1	
2^nd ^quartile	264	19	7.2%	1.78	0.83-3.81
3^rd ^quartile	262	30	11.4%	**2.96**	**1.45-6.04**
4^th ^quartile (richest)	263	41	15.6%	**4.23**	**2.12-8.43**

'Total' indicates the number of households for each factor categories; 'N' is the number of households where modern medicine was present and '%' the corresponding percentage of households.; The odds ratio (OD) with the corresponding 95% confidence interval (95% CI) are given. Statistically significant results are in bold

Other factors increasing the likelihood of medicine being present in the household included household head being literate (14% vs. 7%, p < 0.001), having at least one child under five (14% vs. 8%, p = 0.006), and increasing economic assets (16% vs. 4%, p < 0.001, when comparing the highest and lowest quartiles).

### Malaria episodes in young children

**Figure 1:**
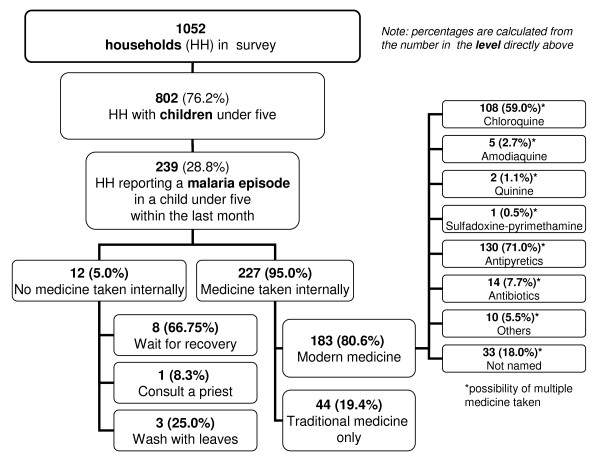
**Treatment of malaria episodes in children under five**.

Out of the 1052 households surveyed, 76% (802/1052) had children under the age of five years (Figure 1). Of these households, 30% (239/802) reported at least one malaria episode in a young child within the last month. There were no differences in the number of self-reported malaria episodes between villages with or without a health centre. While 95% (227/239) of episodes were treated with modern, traditional or mixed medicine, 5% (12/239) of episodes were not treated with medicine taken internally. Treatment consisted either of modern medicine in 72% (172/239), traditional remedies in 18% (44/239) or a mix of both in 5% (11/239) of cases.

Reported malaria symptoms included fever (90%, 216/239), vomiting (46%, 109/239), lack of appetite (21% 50/239), shivering (7%, 16/239) and convulsions (3%, 8/239). Coughing (26%, 62/239) and diarrhoea (24%, 58/239) were also frequently reported.

### First action of caregivers and treatment of malaria episodes

**Figure 2:**
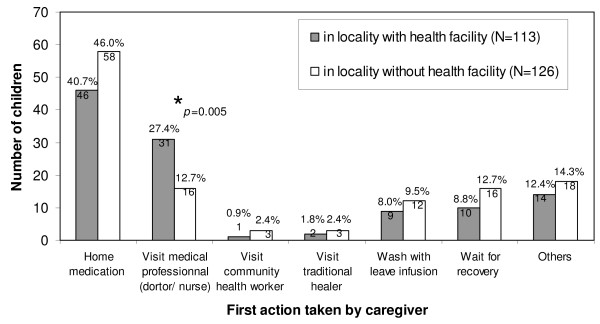
**First action taken by caregiver after appearance of symptoms in children**.

Figure 2 shows the first action taken by parents/caregivers with the occurrence of symptoms in under five children. Home treatment by the caregiver was the most frequent procedure, followed by treatment through a medical professional (nurse or medical doctor). Home treatment was not statistically different in localities with compared to localities without a health facility (41% vs. 46%, p = 0.435). In localities with a health facility compared to localities without one about twice as many children were taken to a medical professional first (27% vs. 13%, p = 0.005). Visiting a CHW or a traditional healer immediately was only reported in a very small number of cases.

77% (183/239) of children with reported malaria received some type of modern medications as first treatment (Figure 1). Administration of multiple modern medication was frequently observed and included chloroquine (45%, 108/239), amodiaquine (2%, 5/239), quinine (1%, 2/239), SP (0.4%, 1/239), anti-pyretics (54%, 130/239), antibiotics (6%, 14/239), and unspecified or other drugs (18%, 43/239). 38% (91/239) of children received a combination of anti-malarial and anti-pyretic. Chloroquine represented 93% (108/116) of all anti-malarials administered. No child received an artemisinin drug or ACT. 14% (33/239) of children received a treatment with modern drugs that could not be remembered or named by the caregiver and might have been an anti-malarial.

**Figure 3:**
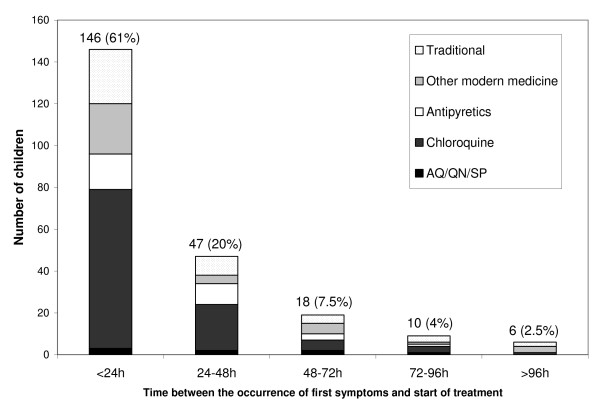
**Time between the occurrence of first symptoms and start of treatment (N = 239)**.

Overall, 61% (146/239) of children received some type of medicine within 24 hours of first symptoms; this figure increased to 81% (193/239) when considering the first 48 hours (Figure 3). Of children being treated during the first 24 hours, 33% (78/239) received modern anti-malarials. In case of home treatment, 58% (60/104) of children took modern anti-malarials within 24 hours. Literacy of the head of the household was the only factor that increased the likelihood that the child be treated within 24 hours (71% vs. 56%, p = 0.02). Other factors such as presence of a health facility in the locality, distance of household to a health facility, age of the child, sex of household's head, ethnic group, or socio-economic status were not associated with early treatment.

### Origin of the medicine used for malaria treatment in young children

**Figure 4:**
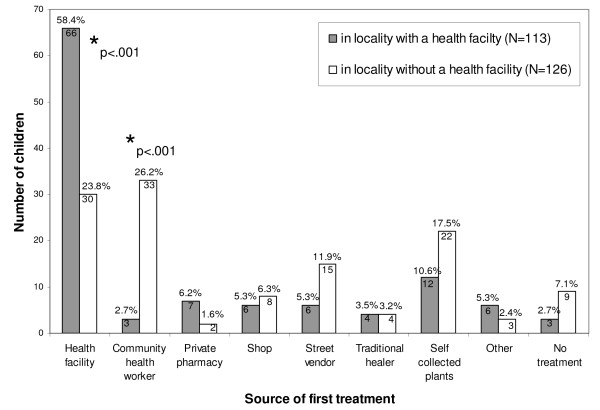
**Source of first treatment**. * indicate statistically significant results.

The decision to buy/use a certain modern treatment was taken by the parents in 61% (112/183) and by a medical doctor, nurse, or pharmacist in 33% (60/183) of the illness episodes. Modern medicine used for first treatment was bought more often from a health facility in localities with a health centre compared to those without (60% vs. 25.6%, p < 0.001) (Figure 4). In contrast, CHW were the main source of modern drugs in localities without a health facility (26% vs. 3%, p < 0.001). A trend indicated that drugs were bought more often at illicit markets (street vendors, markets, shops) in localities without than in localities with a health facility (20% vs. 11%, p = 0.07).

Fifteen percent (33/227) of the drugs used for first treatment were already available in the household at the time of the child's malaria episode and of those 79% (26/33) were modern drugs.

### Second/third treatment and outcome of malaria episodes

Twenty-five children (11%, 25/239) received a second treatment and of these six (3%, 3/239) took a third treatment. No child that had been treated with amodiaquine, SP or quinine as first treatment received a second treatment. However, among the children that received a second treatment, 16 were treated with modern medicine including three taking amodiaquine or SP, and five taking chloroquine. No anti-malarials were given as third treatment.

At survey time the illness was still on-going for 18% (42/239) of the study children, while the majority (82%, 196/239) had recovered. Overall 4% (9/239) of children were hospitalized (seven in a health centre and two in the Nouna district hospital); one child (0.4%) died at the Nouna hospital.

## Discussion

This study provides detailed information on access to and use of anti-malarial drugs from an African area with high malaria transmission intensity. The data from this large household survey are representative for a rural health district of Burkina Faso and can probably be considered comparable to the situation in many rural areas of West Africa. The data were collected in 2006, when ACT had already become first-line treatment for malaria in most of the malaria endemic countries of SSA including Burkina Faso [5,21,27].

Not much information exists on the availability of anti-malarial medicine in households of the endemic areas in SSA. In Tanzania home stocking of anti-malarials in households ranged from 33% at the time of chloroquine to 8% at the time of SP first-line treatment [48]. In the Nouna area it was shown that 14% of households possessed medicines perceived to be effective against malaria, and the availability of more effective anti-malarials was slightly (p = 0.07) associated with the proximity of a health facility. Such households have better access to medicines and may benefit in case of disease. Important determinants of anti-malarial medicine in households identified in this study were the availability of modern health facilities in the community as well as household characteristics, such as literacy of household head, presence of young children, and high socio-economic status. However, how to interpret better availability of modern drugs in households is not clear. The possession of drugs could be beneficial as malaria might be treated earlier, but could also become problematic if this leads to non-adherence with official drug regimens. Thus, further studies on this topic are needed after ACT as first-line treatment - with or without laboratory-based diagnosis - has become fully implemented in the area.

Among the main findings from this study is that the great majority of malaria episodes were still treated with chloroquine, anti-pyretics and traditional medicine, which confirms former findings [27,49] and fits well with the pattern of household availability of anti-malarials found in this study. More effective modern anti-malarials such as amodiaquine, SP or quinine were only used in a small proportion of cases and exclusively in localities with modern health facilities. ACT, despite being the official first-line regimen for malaria treatment since 2005, was not used at all. These findings confirm the still very low ACT coverage in SSA and provide further evidence for the difficulties related to treatment policy changes in malaria endemic countries [21,24,50,51].

Another important finding is that most of the modern anti-malarials originated from governmental health services, with health centres being the main source for purchase of drugs in localities with a health centre and CHW being the main source for purchase of drugs --but interestingly not as point of first reference after the start of symptoms-- in localities without a health centre. In Burkina Faso, CHW were trained and established in villages without a health centre during the 1980s, but have not received much attention or specific support thereafter. In recent years, a number of community-based projects as well as the Ministry of Health have trained or re-trained a certain number of CHW in many villages of Burkina Faso. However, no official document is available on the current status and function of the remaining CHWs in the villages of the Nouna Health District. As there is currently an international revival of the primary health care (PHC) movement [52-56], strengthening the role of CHW in malaria treatment and beyond should be considered as a potential answer to the lack of access to modern health services and the health worker crisis in SSA [15,57-61]. Some promising examples from Asia and Africa regarding the role of CHWs in improving access to ACT have recently been published [62,63]. Apart from being equipped with effective oral anti-malarial regimens, such volunteers may also use pre-referral rectal artesunate in children with severe malaria to reduce mortality [64]. However, such programmes would need to be accompanied by political support from the highest levels as well as by measures to improve the training, motivation and supervision of CHW [9,65-67].

Another finding of this study was that modern anti-malarials were more often purchased from illicit sources, such as shops and markets, in communities without a health centre compared to those with a health centre. In a recent study conducted in the same study area, it was shown that many of the anti-malarials purchased from illicit sources were of substandard quality [34]. This further supports the importance of access to functioning health services including quality-controlled drugs in rural SSA [50,68-70]. Highly subsidized and quality-controlled ACT regimens supported by a global funding mechanism could be one answer to the problem [71,72].

Early access to effective treatment is clearly associated with reduced malaria morbidity and mortality in endemic areas [29,73]. In this study, it was reported that roughly a third of all under five children received treatment with some type of modern anti-malarial drug within 24 hours of the onset of symptoms, and this figure increased to 58% when considering only home treatment. The Abuja declaration stated that at least 60% of malaria cases should have prompt access to affordable and appropriate treatment within 24 hours of the onset of symptoms [74] while more recent targets of the RBM partnership have called for at least 85% of malaria cases in children under five having received ACT treatment within 24 hours of the onset of symptoms [75]. However, even without considering the high resistance of *Plasmodium falciparum *against chloroquine and the non-availability of ACT at the time of the study in the Nouna area [27], these targets have not been reached. This supports similar findings from other SSA areas [36,76-78]. Interestingly, in the Nouna area home treatment with modern anti-malarials was started much more rapidly compared to treatment given by a medical professional. This highlights again the importance of access to modern medicine and points to the likely benefit of reviving the CHW concept in rural SSA [9,66]. Home treatment services using CHW or alternative volunteers such as mother group leaders in collaboration with the nurses of existing health facilities were recently shown to be feasible and effective in increasing treatment coverage in SSA countries including Burkina Faso [9,51,63,79,80].

The finding of this study that subsequent treatments were applied only in cases where chloroquine and not of other modern anti-malarials were used as first treatment supports evidences of the high level of chloroquine resistance in the area [27]. Out of 239 reported febrile disease episodes resembling malaria, nine cases were hospitalized and one child died. As the proportion of true malaria cases amongst these episodes is unknown, it is not possible to estimate cause-specific mortality rates from these figures.

Due to new funding through the Global Fund against AIDS, tuberculosis and malaria, ACT is now available in the Nouna Health District as in all of Burkina Faso since the end of 2007. These drug regimens can be purchased from all governmental health facilities at a subsidized prize, which is however several times above the former prize for chloroquine. Moreover, long distances to rural health centres, transport problems and lack of money to pay for transport, services and opportunity costs will certainly continue to limit early access of young children with malaria to modern health facilities in Burkina Faso, as in most of rural SSA [21,27,30,41,81,82]. Strengthening the role of CHW or other volunteers trained to distribute anti-malarials may thus remain the only short-term solution for increasing access to early effective malaria treatment in rural SSA.

This study has some limitations. Firstly, having been part of a repeated annual cross-sectional household survey in the Nouna Health District in the frame of an ITN trial [46], the study was conducted in the middle of the dry season. Thus, compared to the situation during the rainy season a higher proportion of the reported malaria cases may in fact be fever cases of other origin. However, the rural Nouna study area is holoendemic for malaria [45] and even during the dry season high levels are observed in term of *Plasmodium falciparum *parasites in peripheral blood with 60 to 80% of under-five children being positive by routine blood smear tests and in term of falciparum malaria prevalence, defined as a fever episode plus a blood parasite count greater than 2,000 per microliter and which ranges between 2 to 5% among under-five children (Mueller, unpublished results). Secondly, due to the study design the study populations may not be totally representative for the whole district. Not all participating villages were selected at random and those with a health centre were usually larger than those without a health centre. This could have introduced a bias as the localities with a health facility may differ from those without a health facility in a number of only partly measured socio-demographic and socio-economic parameters. Finally, the findings reported from this study are based on self-reported information on morbidity, health care-seeking behaviour, and drug ownership and use. Such data may be influenced by reporting bias due to imprecise remembering of events, lack of knowledge on specific medicines, or perceived pressure to provide socially acceptable answers.

## Conclusion

In conclusion, access to modern quality health services and effective anti-malarials remains severely limited in rural Burkina Faso as in most of SSA. Given the continuous financial and health worker crisis, innovative systems of home treatment based on locally appropriate primary health care strategies such as revival of CHW strategies or training and support of other community volunteers, which needs to include the large-scale provision of effective anti-malarial regimens, such as ACT, should be piloted more vigorously in the malaria endemic rural areas of SSA. Such pilot schemes need of course to be accompanied by well-designed applied research. Thirty years after Alma Ata, it is urgent time to roll out comprehensive primary health care in SSA.

## Competing interests

The authors declare that they have no competing interests.

## Authors' contributions

MT contributed to the conception and the design of the study, developed the questionnaire, collected the data, and contributed to the analysis and interpretation of the data and to the writing of the paper. VRL contributed to the analysis and interpretation of the data and wrote the paper. MY contributed to the interpretation of the data and to the writing the paper. MDA participated on the conception and the design of the study, developed the questionnaire, and contributed to the interpretation of the data and to writing the paper. CB contributed to the interpretation of the data and to writing the paper. AS contributed to the conception and the design of the study, the interpretation of the data and to writing the paper. OM initiated the conception and design of the study, contributed to the development of the questionnaire and the interpretation of the data, and wrote the paper. AJ contributed to the conception and the design of the study, to the interpretation of the data and to writing of the paper. All authors read and approved the final manuscript.

## Supplementary Material

Additional file 1 **Map of health facilities in Nouna Health District**. Map of health facilities in Nouna Health District. Red crosses indicate villages with a health facility and 'H' the town of Nouna with the District Hospital. Click here for file

Additional file 2 **Survey questionnaire**. Survey questionnaire used for the study, in French. Click here for file
