# Supplementary figures and images for: Access to malaria treatment in young children of rural Burkina Faso
Source: Malar J. 2009 Nov 24;8:266. doi: 10.1186/1475-2875-8-266 (PMC2790466; doi:10.1186/1475-2875-8-266)

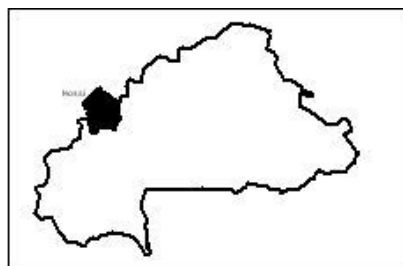

# FORMATIONS SANITAIRES DU DISTRICT DE NOUNA

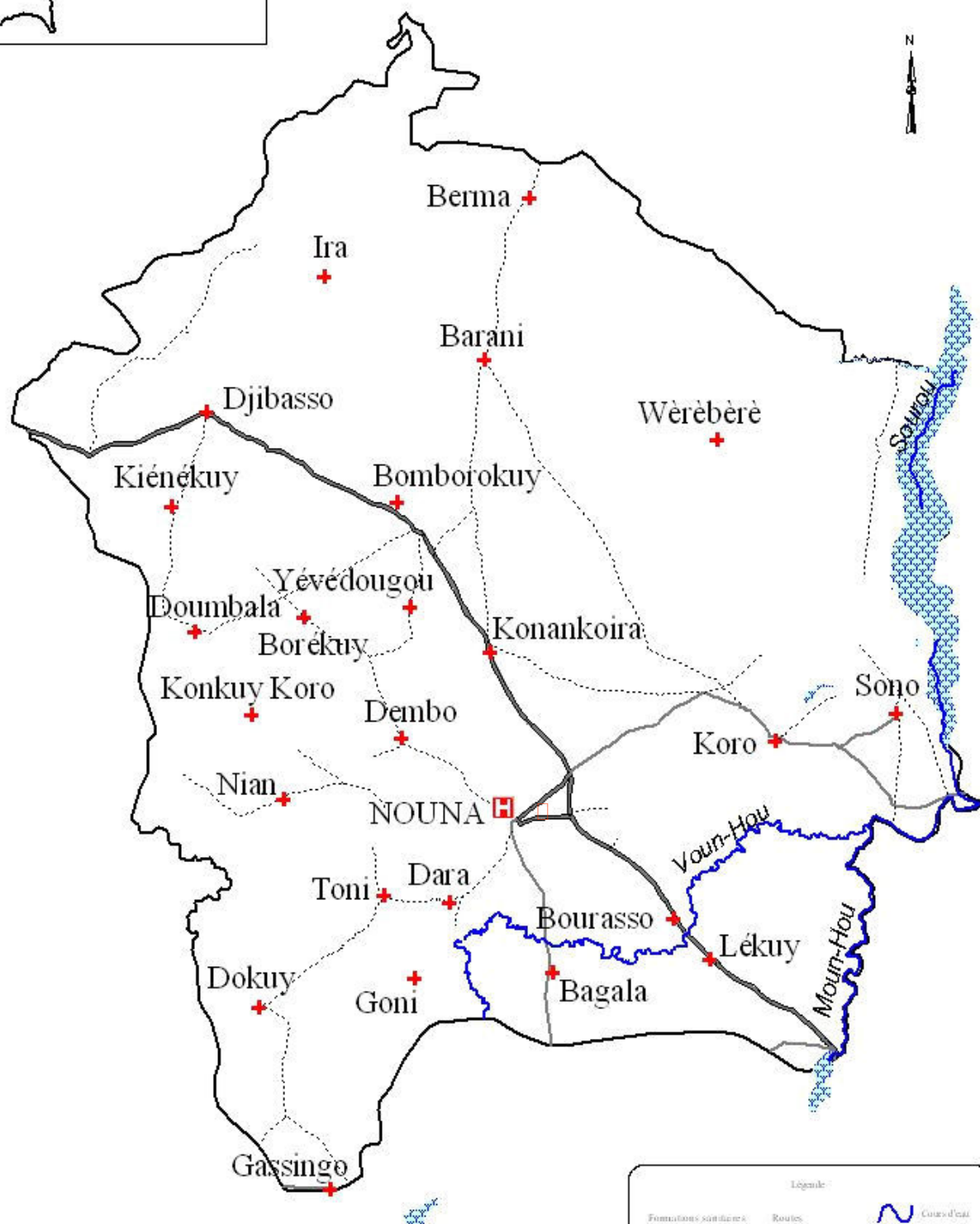

Supplement: Additional file 1 — Map of health facilities in Nouna Health District. Map of health facilities in Nouna Health District. Red crosses indicate villages with a health facility and 'H' the town of Nouna with the District Hospital. [file 1475-2875-8-266-S1.PDF]
