# Supplementary material for: Access to malaria treatment in young children of rural Burkina Faso
Source: Malar J. 2009 Nov 24;8:266. doi: 10.1186/1475-2875-8-266 (PMC2790466; doi:10.1186/1475-2875-8-266)
Supplement: Additional file 2 — Survey questionnaire. Survey questionnaire used for the study, in French. [file 1475-2875-8-266-S2.PDF]

Ne réponde pas = 97
Ne sait pas = 98
Ne s'applique pas = 99

ID ménage : □□□ □□□ □□

Section 7

Maintenant je voudrais vous poser quelques questions concernant de traitement de paludisme/soumaya dans votre ménage.

93. Avez-vous présentement des médicaments contre le paludisme/ soumaya disponibles chez vous ? …………………………………………………………………...….**93.**□□

Codes réponse 93:

[1] **Oui**
[2] **Non**

Si OUI, passez à la question 94.
Si NON, passez à la question 96.

Si vous avez des médicaments, précisez lesquelles?
En ce moment, l'enquêteur se fait montrer chaque médicament.

	**94. nom du médicament**<br>*Soyez très attentif et notez le nom des médicaments correctement! En cas ou le nom ne peut pas être trouvé et il y a juste des médicaments sans emballage, décrivez le produit si précisément que possible.*<br>*Par exemple*<br>*Comprimés blancs, Sirop rouge, Infusion,….*<br><br>*S'il y a moins de 4 médicaments disponibles mettez le mot « **rien** » dans chaque case qui reste.*	**95. D`où aviez-vous eu ces médicaments ?**<br>[1] **l'hôpital**<br>[2] **CSPS**<br>[3] **agent santé villageois**<br>[4] **pharmacie privée**<br>[5] **boutique**<br>[6] **marchands ambulants**<br>[7] **tradi-praticien / guérisseur**<br>[8] **cueilli/ collecté au brousse**<br>[9] **autre**____________________
**94.a**		**95.a** □□
**94.b**		**95.b** □□
**94.c**		**95.c** □□
**94.d**		**95.d** □□

Ne réponde pas = 97
Ne sait pas = 98
Ne s’applique pas = 99

ID ménage : □□□ □□□ □□

Maintenant je voudrais que vous vous rappeliez du dernier cas du paludisme/ soumaya depuis le dernier mois d`un enfant moins des 5 ans dans votre ménage.

96. Est ce qu'il y a eu un cas de paludisme/soumaya chez un de vos enfants moins de 5 ans dans le dernier mois? ……………………………………………………**96.** □□

Codes réponse 96:

[1] **Oui**
[2] **Non**

Si OUI, passez à la question 97.
Si NON, terminez et remercier pour les repense.

Si il y a plusieurs cas du paludisme/ soumaya dans le dernier mois je me intéresse du cas qui a été le plus proche de cette enquête. J’aimerais poser les questions suivent à la mère ou autre personne qui s’occupe de l’enfant.

97. S’il y a un cas du paludisme/ soumaya, précisez l'age de l'enfant?...............**97.** □□

Codes réponse 97:

[1] **0- 6 mois**
[2] **7-11 mois**
[3] **1- 3 ans**
[4] **4- 5 ans**

98. Quels ont été les symptômes principaux? **98.a** □□ **98.b** □□ **98.c** □□

……………………………………………………………………………**98.d** □□

Codes réponse 98: Question a Choix Multiple !

[1] **fièvre**
[2] **frissons différent de convulsion**
[3] **vomissement**
[4] **diarrhée**
[5] **manque ou (perte) d`appétit**
[6] **toux**
[7] **convulsions**
[8] **maux de tête**
[9] **fatigue général**
[10] **autre**________________________

Indiquez les symptômes à l´ordre chronologique !

Ne réponde pas = 97
Ne sait pas = 98
Ne s’applique pas = 99

ID ménage : □□□ □□□ □□

99. A l'apparition des premiers signes, qu'est-ce que vous avez fait premièrement ? …………………………………………………………………………………..**99.** □□

Codes réponse 99:

[1] **j´ai donné les médicament tout de suite**
[2] **j´ai attendu que la malade s`améliore seule (spontanément)**
[3] **j´ ai consulté un agent de santé (un médecin/ un infirmier)**
[4] **un agent de santé communautaire**
[5] **un tradi-particien / un guérisseur**
[6] **j´ ai lavé l’enfant avec des feuilles bouillies**
[7] **autres**____

100. Avez-vous utilisez des médicaments pour le traitement durant le reste de temps de la maladie?.. **100.** □□

Codes réponse 100:

[1] **Oui**
[2] **Non**

Si OUI, passez à la question 102.
Si NON, passez à la question 101.

101. Si non, que est-ce que vous avez fait ?.. **101.** □□

Codes réponse 101:

[1] **j´ai attendu que le malade s`améliore seule (spontanément)**
[2] **guérisseur du village**
[3] **prière**
[4] **scarification**
[5] **laver avec des feuilles bouillies**
[6] **autres**____

passez à la question 112.

Ne réponde pas = 97 ID ménage : □□□ □□□ □□
Ne sait pas = 98
Ne s'applique pas = 99

Posez cette question à tous ceux qu'ont répondu soit [1] à la Q 99 soit [1] à la Q 100.

Q: Quelles sortes des médicaments avez-vous utilisé pour le traitement de ce cas de paludisme/ soumaya ? *Indiquez touts les médicaments (médicaments modernes et traditionnels) on a donné á l´enfant. Préciser l´ordre chronologique au file du temps. Si l´enfant a pris les médicaments ensemble indiquez les médicaments dans la même case.*

	**En première intention P1**	**En deuxième intention P2**	**En troisième intention P3**
**102. nom du médicament**<br>*Où une explication des médicaments si l'on ne rappelle pas du nom Par exemple Comprimés blancs, Sirop rouge, Infusion,…. Indiquez « **rien** » sur chaque ligne qui reste.*	**a.**________<br>**b.**________<br>**c.**________	**a.**________<br>**b.**________<br>**c.**________	**a.**________<br>**b.**________<br>**c.**________
**103.** Combien de temps s´est écoulé entre les premiers symptômes et le début de traitement avec ces médicaments ?<br>[1] **< 1 jour**<br>[2] **1 jour**<br>[3] **2 jour**<br>[4] **3 jours**<br>[5] **> 4 jours**	□□	□□	□□
**104.** Qui a pris la décision de donner les médicaments à l'enfant ?<br>Codes :<br>[1] **agent de santé (un médecin/un infirmier)**<br>[2] **agent de santé communautaire**<br>[3] **un tradi-praticien / guérisseur**<br>[4] **un pharmacien**<br>[5] **l´un des parents**<br>[6] **autre______**	□□	□□	□□

Ne réponde pas = 97

Ne sait pas = 98

Ne s'applique pas = 99

ID ménage : ☐☐☐ ☐☐☐ ☐☐

	En première intention	En deuxième intention	En troisième intention
**105.** Est-ce que ces médicaments étaient déjà disponibles dans le ménage ?<br>[1] **Oui**<br>[2] **Non**	**a.** ☐☐<br>**b.** ☐☐<br>**c.** ☐☐	**a.** ☐☐<br>**b.** ☐☐<br>**c.** ☐☐	**a.** ☐☐<br>**b.** ☐☐<br>**c.** ☐☐
**106.** Où avez-vous eu ces médicaments?<br>Codes :<br>[1] **l´hôpital**<br>[2] **CSPS**<br>[3] **agent santé villageois**<br>[4] **pharmacie privée**<br>[5] **boutique**<br>[6] **marchands ambulants**<br>[7] **tradi-praticien / guérisseur**<br>[8] **cueille/ collecté au brousse**<br>[9] **autre________**<br><br>***Si [8] passez à Q109***	**a.** ☐☐<br>**b.** ☐☐<br>**c.** ☐☐	**a.** ☐☐<br>**b.** ☐☐<br>**c.** ☐☐	**a.** ☐☐<br>**b.** ☐☐<br>**c.** ☐☐
**107.** Qui a payé ces médicaments ?<br>[1] **chef du ménage**<br>[2] **la mère**<br>[3] **la maratre**<br>[4] **autre membre du famille**<br>[5] **pas payé**	**a.** ☐☐<br>**b.** ☐☐<br>**c.** ☐☐	**a.** ☐☐<br>**b.** ☐☐<br>**c.** ☐☐	**a.** ☐☐<br>**b.** ☐☐<br>**c.** ☐☐
**108.** Avez-vous acheté les médicaments sur ordonnance?<br>[1] **Oui**<br>[2] **Non**	**a.** ☐☐<br>**b.** ☐☐<br>**c.** ☐☐	**a.** ☐☐<br>**b.** ☐☐<br>**c.** ☐☐	**a.** ☐☐<br>**b.** ☐☐<br>**c.** ☐☐
**109.** Pendant combien des jours avez-vous donné les médicaments ?<br>***Un jour = 1***<br>***Deux jours=2, etc.***	**a.** ☐☐<br>**b.** ☐☐<br>**c.** ☐☐	**a.** ☐☐<br>**b.** ☐☐<br>**c.** ☐☐	**a.** ☐☐<br>**b.** ☐☐<br>**c.** ☐☐

Ne réponde pas = 97
Ne sait pas = 98
Ne s'applique pas = 99

ID ménage : □□□ □□□ □□

110. Est-ce que l'enfant a été hospitalisé au cours de ce cas du paludisme/ soumaya ?

……………………………………………………………………………..**110.** □□

Codes réponse 110:

[1] **Oui**
[2] **Non**

Si OUI, passez à la question 111.
Si NON, passez à la question 112.

111. Si oui, préciser le lieu ? …………………………………………………….**111.** □□

Codes réponse 111:

[1] **hôpital**
[2] **CSPS**

112. Qu'est ce qui était le résultat de traitement de l'enfant?........................... **112.** □□

Codes réponse 112:

[1] **guéri**
[2] **maladie toujours en cours**
[3] **aggravé**
[4] **décédé**
